# Municipal deprivation and cardiometabolic outcomes in Mexican adults: findings from ENSANUT 2021–2023

**DOI:** 10.1186/s12939-025-02754-2

**Published:** 2026-01-10

**Authors:** David Contreras-Loya, Martín Romero-Martínez, Paola Abril Campos-Rivera, Héctor Arreola-Ornelas, Linda Morales-Juárez

**Affiliations:** 1 Tecnologico de Monterrey, Institute for Obesity Research, Unit of Health Public Policy, Mexico City, Mexico; 2 Tecnologico de Monterrey, Escuela de Gobierno y Transformación Pública, Mexico City, Mexico; 3 Instituto Nacional de Salud Pública, Centro de Investigación en Evaluación y Encuestas, Cuernavaca, Mexico; 4 Tecnologico de Monterrey, Escuela de Ciencias Sociales y Gobierno, Mexico City, Mexico; 5 Universidad Nacional Autónoma de México, Public Health Department, Mexico City, Mexico

**Keywords:** Health inequities, Diabetes cascade, Municipal deprivation, Mexico, Structural determinants, Metabolic control

## Abstract

**Background:**

Cardiometabolic diseases are rising rapidly in low- and middle-income countries. Managing them requires a full cascade of care: early detection, treatment, and long-term control. Yet in Mexico, many adults are not reached by effective care, especially in poorer municipalities where health services are scarce. These local inequities mean that the municipality of residence can strongly determine whether their condition is detected, treated, or controlled.

**Methods:**

We used the Mexican National Health and Nutrition Survey 2021–2023 (ENSANUT), a sequence of independent national probabilistic, nationally and regional representative surveys with a total sample size of 32,087 adults (20 + years old). Municipal deprivation was assessed using the Density-Independent Social Lag Index (DISLI). Primary outcomes were diabetes identification, treatment, and glycemic control (the diabetes care cascade), along with indicators for hypertension, adiposity, metabolic syndrome, dyslipidemia, and kidney function. We used survey-weighted descriptive statistics and regression analysis to quantify disparities in outcomes across levels of municipal deprivation.

**Results:**

We found that screening participation and disease prevalence for diabetes, hypertension and dyslipidemia varied little among Mexican municipalities. However, two separate gradients were apparent: a steep gradient in diabetes control and an inverse gradient in metabolic risk. Adjusted estimates for diabetes control (HbA1c < 7%) dropped from 31.8% in the least to 13.7% in the most deprived areas. By contrast, the wealthier municipalities had higher levels of obesity and metabolic syndrome. Meanwhile, control of hypertension was equally poor (33% of treated cases) across all strata.

**Conclusions:**

Deprivation had no impact on case finding at the local level, but it was related to significantly worse glycemic control. Those from poor areas were much less likely to achieve glycemic targets, reflecting structural care deficits. Higher obesity and metabolic risk in wealthier settings, however, point to the need for prevention efforts to focus on urban obesogenic environments. To narrow the inequity gap, proportionate universalism is needed: greater support for managing diabetes in disadvantaged towns and broader prevention strategies in advantaged ones.

**Supplementary Information:**

The online version contains supplementary material available at 10.1186/s12939-025-02754-2.

## Introduction

Cardiometabolic risk factors including Type 2 diabetes mellitus (T2D), hypertension, dyslipidemia, and early kidney impairment constitute a convergent driver of cardiovascular and renal disease worldwide [1, 2]. Their prevalence is rising fastest in low- and middle-income countries (LMICs), where demographic transitions and urbanization amplify the exposure to obesogenic environments and constrain access to high quality healthcare [3, 4].

Population ageing in many LMICs is likely to intensify these challenges. In Mexico for example, 11% of the population was aged 60 + in 2020, and this share is expected to approach one quarter by 2050 [5]. While this shift reflects gains in survival, is also increases pressure on health systems to prevent and manage chronic non-communicable diseases. In response, many systems have adopted a “cascade of care” approach to track how patients progress through sequential steps of care [6, 7]. Originally developed for HIV/AIDS treatment [8], the cascade typically describes movement from screening and diagnosis to treatment initiation, ongoing adherence, and disease control. Applied to metabolic health, this framework helps identify where patients disengage or face barriers across the pathway, emphazising the need solid for effective support at every stage [9].

Effective management of these conditions demands sequential engagement at four stages: timely detection, linkage to care, sustained pharmacological and behavioral treatment, and attainment of biomarker-specific control targets [10, 11]. This continuum, hereafter termed the metabolic health service cascade, exhibits substantial attrition: large shares of adults remain undiagnosed, many of those do not initiate or adhere to treatment, and only a minority achieve simultaneous control of the relevant biomarkers. These drop-offs accelerate the onset of preventable complications and premature mortality [12].

Gaps along the cascade are not randomly distributed. Variations that are unnecessary, avoidable, or excessive constitute health inequities and therefore warrant rigorous quantification and policy action [13, 14]. Evidence shows that both individual socioeconomic status (SES) and the broader socioeconomic context, namely neighborhood resources, labor-market opportunities, and spatial distribution of health services, shape these inequalities. Individual-level analyses can mask structural determinants of both risk exposure and access to effective care [15].

Municipality-based (area-based) deprivation has emerged as a powerful structural determinant of cardiometabolic disorders and is typically quantified by composite indices of poverty, education, housing quality, and access to public services [16]. Its adverse effects accumulate across the life course and are partly independent of individual or household SES. Material deprivation, psychosocial stress, reduced walkability, and limited access to nutritious foods act through complementary pathways, increasing insulin resistance, blood pressure, circulating lipids, and renal load, thus impeding effective management of chronic diseases [16, 17]. Moreover, attempts to modify behaviors one person at a time are often difficult to sustain; when unhealthy behaviors are widespread, a purely one-to-one biomedical model becomes an inefficient strategy for population-level control of metabolic risk [18].

Mexico exemplifies these global patterns in amplified form. Nationally representative 2022 data indicate that 16% of adults have T2D; hypertension prevalence ranges from 29% under Eighth Joint National Committee (JNC 8) to 48% under the 2017 of the American College of Cardiology/American Heart Association (ACC/AHA) thresholds; 30% report a prior diagnosis of hypercholesterolemia; and approximately 1% report chronic kidney disease [19, 20]. Among adults with T2D, one third of cases remain undiagnosed and less than half of those previously diagnosed achieve glycemic control; 65% of those with hypertension are unaware of their condition and 34% of those previously diagnosed reach blood pressure targets; among those with high blood lipids, 60% receive lipid-lowering medication yet barely 30% achieve the recommended lipid concentrations; awareness and treatment rates of early-stage kidney disease remain below one third [21]. These cascade failures translate into excess cardiovascular and renal mortality and disability.

Disparities in Mexico are pronounced, as adults without social security coverage and residents in poorer or rural municipalities historically face lower coverage for health services, higher complication rates, and greater mortality [15, 22]. Although geographic and socioeconomic gradients in diabetes prevalence and mortality are well documented, much less is known about how municipality-level deprivation shapes each stage of the broader metabolic health service cascade [23, 24]. Prior studies have focused on isolated outcomes or individual risk factors, overlooking whether structural deprivation amplifies attrition from diagnosis to control [1, 25].

We address this gap for Mexico using a sequence of nationally representative surveys and biomarker data collected between 2021 and 2023. We test the hypothesis that adults residing in more deprived municipalities are less likely to be diagnosed, treated, and controlled for each cardiometabolic risk factor than adults in more affluent municipalities, after accounting for sociodemographic differences in individual and household characteristics. Identifying the cascade stages most affected by municipal deprivation will inform policies that move beyond individual level counseling toward structural interventions capable of reducing inequities in metabolic health.

## Methods

### Study design and data sources

We quantified the association between municipality-level deprivation and metabolic health with a cross-sectional analysis using data from the Mexican National Health and Nutrition Survey (ENSANUT Continua) 2021–2023 [26]. ENSANUT is a population-based probabilistic survey that employs a multi-stage, stratified cluster sampling design, yielding representative data of the population. Data were collected via face-to-face interviews, standardized questionnaires, and biometric measurements. A probabilistic subsample of participants provided blood samples for laboratory assays to assess biochemical health indicators. All respondents consented to participate, and the ENSANUT survey protocol was approved by the Research Ethics Committee of the Mexican National Institute of Public Health. We joined data from the 2021, 2022, and 2023 survey rounds to increase statistical power for the inferences relevant to this study.

### Study population

The study population consisted of adult aged 20 years and older that lived in México from 2021 to 2023. We excluded women who reported only gestational diabetes and any participants who were pregnant at the time of survey, as their diabetes care needs differ. Venous blood was collected from a biomarker subsample (*N* = 5,678), allowing measurement of glycated hemoglobin (HbA1c), fasting glucose, HDL-C, LDL-C, triglycerides, and serum creatinine; these data enabled the evaluation of biomarker-based detection and control across the metabolic health service cascade.

### Outcome measures

Outcome variables were specified to parallel the detection–treatment–control sequence of the metabolic health service cascade. From the pooled data, we identified all individuals with T2D, hypertension, dyslipidemia, and kidney failure/chronic kidney (Table 1).

**Table 1:** Outcome measures, operational definitions, and corresponding stages in the metabolic health service cascade

Cascade element / Outcome	Operationalization
**Screening/**	
Detection of Type 2 Diabetes (T2D) (screening)	Reports having been tested or screened for diabetes in the last 12 months.
**Diagnosis / Total prevalence/**	
Prediabetes (HbA1c-defined)	HbA1c 5.7–6.4% in the biomarker subsample.
T2D (biochemical (HbA1c) or self-reported; total prevalence)	HbA1c ≥ 6.5% in the biomarker subsample or reports a previous physician diagnosis of diabetes.
Elevated blood pressure (2017 ACC/AHA)^1^	Mean systolic blood pressure ≥ 130 mmHg or mean diastolic blood pressure ≥ 80 mmHg across three seated readings.
Hypertriglyceridemia	Triglycerides ≥ 150 mg/dL or lipid‑lowering therapy.
Low HDL‑cholesterol	HDL‑cholesterol < 40 mg/dL (men) or < 50 mg/dL (women).
High LDL‑cholesterol	LDL‑cholesterol ≥ 100 mg/dL.
High total cholesterol	Total cholesterol ≥ 200 mg/dL.
**Diagnosis / Awareness /**	
T2D (self‑reported)	Answers “yes” to “Has any doctor told you that you have diabetes or high blood sugar?”.
Hypertension (self‑reported)	Answers “yes” to “Has any doctor told you that you have high blood pressure or hypertension?”.
Dyslipidemia (self-reported)	Reports that a doctor has told them they have high cholesterol.
**Disease control/**	
Controlled T2D (HbA1c, %)	Among individuals with T2D, HbA1c < 7.0%.
Mean HbA1c among individuals with T2D	Mean HbA1c among participants classified as having T2D.
Mean fasting glucose among individuals with T2D	Mean fasting plasma glucose among participants classified as having T2D.
Mean fasting glucose among individuals with uncontrolled T2D	Mean fasting plasma glucose among participants with T2D who do not meet the HbA1c control threshold.
Controlled hypertension	Among individuals with hypertension, mean systolic blood pressure < 130 mmHg and mean diastolic blood pressure < 80 mmHg (2017 ACC/AHA) [27].
**Other Outcomes/**	
eGFR categories (CKD-EPI stages G1–G5)^2^	eGFR derived from serum creatinine using the CKD‑EPI creatinine equation, categorized as G1 (≥ 90), G2 (60–89.9), G3a (45–59.9), G3b (30–44.9), G4 (15–29.9), or G5 (< 15 mL/min/1.73 m²) [28, 29].
Impaired kidney function	eGFR < 60 mL/min/1.73 m² (CKD‑EPI stages G3a–G5).
Overweight + obesity (BMI-defined)^3^	BMI ≥ 25 kg/m².
Obesity (BMI-defined)	BMI ≥ 30 kg/m².
Abdominal obesity	Waist circumference ≥ 80 cm in women or ≥ 90 cm in men.
Metabolic syndrome (IDF criteria)^4^	Abdominal obesity plus at least two of the following components: raised triglycerides, reduced HDL‑cholesterol, raised blood pressure, or elevated fasting glucose (≥ 100 mg/dL) [30].

Notes: (1) ACC (American College of Cardiology); AHA (American Heart Association); (2) estimated glomerular filtration rate (eGFR); (3) body-mass index (BMI); (4) IDF (International Diabetes Federation)

### Exposure: Municipality-level deprivation

We measured contextual deprivation using the density-independent Social Lag Index (DISLI), an adaptation of CONEVAL’s [31] national Social Lag Index (SLI) that removes its correlation with population density. Following the published methods [32], we constructed a density-independent social lag index (DISLI) by regressing the municipal SLI on the log of population density ($SL{I_m} = \alpha + \beta \cdot log(densit{y_m}) + {\varepsilon _m}$) and defining DISLI as the standardized Ordinary Least Squares (OLS) residual ($$\:\epsilon\\hat :$$). By construction, DISLI is orthogonal to population density and captures the component of deprivation unexplained by density; higher values indicate greater deprivation net of density. We standardized residuals to mean 0 and SD 1 using the distribution across municipalities. As a check, DISLI was near-uncorrelated with log density and remained strongly correlated with canonical deprivation components. The underlying SLI is built from ten census indicators covering deficits in education, housing quality, basic services, healthcare access, and household assets. We linked each ENSANUT participant to the 2020 municipal DISLI of their residence.

To create analytically balanced strata while preserving the method’s data-driven cut points, we applied the Dalenius–Hodges [33] cumulative square root frequency algorithm to the municipal DISLI distribution but retained four ordered strata rather than the original five. Collapsing the two lowest and two highest strata ensured adequate numbers of observations per group without distorting the monotonic gradient of disadvantage. The resulting strata (lowest, lower-middle, upper-middle, and highest deprivation) were treated as an ordinal exposure (see Supplementary Methods S1 for full details).

### Covariates

The analyses adjusted for a consistent set of individual-, household-, and municipality-level covariates. Socioeconomic position was proxied by the ENSANUT wellbeing (*bienestar*) index, grouped into tertiles, and by an indicator of current labor-market participation (employed or actively seeking work). Demographic covariates comprise age (years, continuous), sex (female = 1), and civil status (married; cohabiting; widowed; not in a union). Educational attainment was classified as basic, lower-secondary, upper-secondary/high-school, and tertiary. Health-related behavior is captured by a binary indicator for current smoking status. Household composition is considered vulnerable when it has at least one child aged < 5 years or one adult aged ≥ 65 years. Access to social protection is captured by a binary indicator for social security coverage. This covariate was coded as “yes” if the respondent was covered by any federal or state social security health system, and “no” otherwise. Social security institutions considered include IMSS, ISSSTE, Petróleos Mexicanos (Pemex) health services, the Secretariat of National Defense (SEDENA) health system, and other analogous public systems (for example, those covering other branches of the armed forces or state-level social security programs with similar coverage). To account for broad spatial heterogeneity, we included dummy variables for eight macro regions: Pacific-North, Border, Pacific-Central, North-Central, Central, Ciudad/Estado de México, South-Pacific, and Yucatán Peninsula. Finally, supply-side context is characterized by three continuous municipality-level metrics: the rate of public and private medical consultation rooms per 10,000 population and the rate of general physicians per 1,000 [34, 35].

### Statistical analysis

All statistical analyses accounted for ENSANUT’s complex sampling design, incorporating primary sampling units, stratification, and the appropriate survey weights; subsample weights were applied for biomarker-based indicators. Weight of individual surveys were divided by 4 to make the sum of weights equal to the average over the period 2021 − 2014, of the number of adults 20 + years old in México. Municipality-level deprivation was summarized using the Density-Independent Social Lag Index (DISLI), categorized into four ordered levels of deprivation as detailed in the Supplementary Methods S1. We began by describing the characteristics of the study population across the four levels of municipality-level deprivation, using means for continuous variables and proportions for categorical variables, and assessed differences across groups via linear trends using Wald tests that modeled the ordinal deprivation variable as continuous. We then estimated the unadjusted prevalence or mean of each outcome by deprivation group using the appropriate survey weights.

For our main analyses, we fitted linear probability models for binary outcomes, linear regression models for continuous outcomes and obtained robust standard errors to account for heteroskedasticity. Each model included municipality-level deprivation as the primary independent variable (treated as categorical, with the lowest deprivation category as the reference) and adjusted for the full covariate set detailed in the Covariates subsection. Survey year (2021, 2022, or 2023) was included as a fixed effect in all models.

We opted for linear probability models (instead of logistic regression) for binary outcomes to facilitate explanation of effects as absolute probability differences (percentage-point changes) and because survey-weighted predictive margins are straightforward to obtain under this approach. Sensitivity checks using logit regression yielded only minimal differences in standard errors and identical prevalence estimates, supporting the linear specification. For interpretation, we used predictive margins to report adjusted outcome levels by deprivation group, facilitating comparisons in absolute terms (e.g., adjusted prevalence of disease control across groups).

The derivation and categorization of the DISLI were implemented in the R version 2025.05.0 + 496 (base lm function for linear regression and the *stratification* package), as described in the Methodological Supplementary Methods S1. All models were estimated in Stata/SE version 17.0 using the *svy* suite, the *margins* command, and all hypothesis tests were two-tailed with a significance level of α = 0.05. Confidence intervals at 95% are reported for all key estimates. Some strata contained small cell counts, which may yield imprecise estimates; results for low prevalence outcomes should be interpreted with caution.

## Results

At the individual level, outcomes were compared across four categories of municipality deprivation of residence (DISLI strata) using ENSANUT 2021–2023. After weighting to the national adult population (≥20 years), the ENSANUT 2021–2023 analytic sample of 32,087 adults (mean age 44.7 years; 52% women) represented approximately 85 million individuals distributed across the four municipal deprivation strata. Although DISLI and the original Social Lag Index remained correlated (Pearson $\:r=0.77$; Kendall $\:{\tau\:}_{b}=0.67$), removing population density from the original score re-ordered 38% of municipalities. High-density urban localities in Ciudad de México, Estado de México, and Jalisco shifted two or three deprivation strata upward, while several rural municipalities in Guerrero, Puebla, and Oaxaca moved toward lower downward. The state composition of these reclassifications, showing the share of municipalities within each shift category that belong to each state (column percentages, with each category shift summing to 100%) is presented in Supplementary Table S1. Therefore, the gradients reported here capture municipal socioeconomic conditions independent of population density.

We present unadjusted prevalences of all cardiometabolic outcomes by municipal deprivation level in Supplementary Table S2.

Sociodemographic gradients followed the new classification: the share with elementary school ranged from 23.4% in low deprivation municipalities to 33.5% in high deprivation municipalities, as did the percentage of households in the bottom tertile of wellbeing index (22.5% to 45.8%). In contrast, social security coverage increased with deprivation (45.3% to 63.1%). The density of private clinics declined as DISLI increased (11.1 per 100,000 in the low deprivation stratum vs. 8.8 in the highest deprivation stratum); public clinics density also decreased with increasing deprivation (from 21.5 to 11.9 per 100,000) except for the least deprived group of municipalities, where density was lowest at 9.7 (Table 2).

**Table 2:** Sociodemographic characteristics by Density-Independent Social Lag Index (DISLI) strata among Mexican adults, ENSANUT 2021–2023

	Density-Independent Social Lag Index (DISLI) strata										
	Total		Lowest		Lower-middle		Upper-middle		Highest		
Sample Size (N)	32,087		20,647		7,285		2,641		1,514		
Expanded N (Millions)	*85.4*		*50.2*		*19.7*		*10.4*		*5.1*		
	**Estimate**	**CI 95%**†	**Estimate**	**CI 95%**	**Estimate**	**CI 95%**	**Estimate**	**CI 95%**	**Estimate**	**CI 95%**	P Trend*
Age (mean)	44.7	44.3–45.0	44.2	43.8–44.7	44.8	44.2–45.5	46.1	45.3–47.0	45.3	43.4–47.1	< 0.01
Women	52.2	51.1–53.2	52.5	51.2–53.8	51.6	49.4–53.9	51.3	48.3–54.3	52.7	49.0-56.4	0.61
Rural area	21.1	17.8–24.8	13.7	10.6–17.5	32.6	24.5–41.8	30.5	20.7–42.4	30.7	14.4–53.9	< 0.001
Urban area	30.3	26.2–34.7	31.3	26.2–37.0	39.0	29.9–49.0	21.1	11.7–35.1	3.8	1.1–12.3	< 0.01
Metropolitan area	48.6	44.2–53.0	55.0	49.4–60.5	28.4	21.3–36.9	48.4	36.1–60.8	65.4	43.5–82.3	0.38
Married	35.5	34.6–36.5	36.2	35.0-37.3	36.7	34.4–39.0	32.2	28.8–35.6	31.4	27.9–34.8	< 0.01
Cohabiting	19.8	18.8–20.7	18.2	17.0-19.4	21.8	20.1–23.5	22.4	19.6–25.2	21.6	17.2–26.0	< 0.01
Not in a union	35.7	34.7–36.7	37	35.9–38.2	31.9	29.8–34.1	36	33.0-39.1	36.8	31.9–41.6	0.22
Widowed	9.0	8.5–9.5	8.6	8.0-9.2	9.5	8.3–10.7	9.4	7.7–11.0	10.3	7.7–12.9	0.06
Elementary school	27.6	26.2–29.1	23.4	21.7–25.2	34	31.5–36.4	32.9	29.1–36.6	33.5	27.8–39.2	< 0.001
Secondary	28	27.0–29.0	28.8	27.4–30.1	28.3	26.4–30.2	24.5	21.6–27.3	26.3	22.4–30.1	0.02
High school	23.5	22.5–24.6	24.9	23.4–26.4	21	19.3–22.7	22.8	20.3–25.3	21.2	16.7–25.6	0.02
Postgraduate	20.9	19.4–22.3	22.9	20.9–24.9	16.7	14.6–18.8	19.8	15.1–24.5	19.1	15.2–23.0	0.02
Working	61.7	60.6–62.9	62.9	61.3–64.4	59.3	57.1–61.5	60.9	58.0-63.7	62.2	59.3–65.1	0.13
Vulnerable household	28.2	26.7–29.8	26.2	24.5–28.0	32.8	29.4–36.2	29.1	24.7–33.4	28.5	21.6–35.3	0.15
Never smoker	64.7	63.5–65.8	62.5	61.1–63.8	68.2	66.0-70.3	67.9	64.8–70.9	66.3	59.8–72.9	< 0.01
Former smoker	16.7	16.0-17.5	17.2	16.2–18.2	15.2	13.8–16.7	16.9	14.8–19.1	17.7	13.9–21.4	0.69
Current smoker	18.1	17.2–19.1	19.9	18.6–21.2	15.9	14.4–17.5	14.7	12.4–17.1	15.8	12.1–19.5	< 0.001
Low well-being (household)	32.1	29.4–34.7	22.5	20.3–24.6	46.4	40.3–52.5	44.2	36.9–51.5	45.8	32.1–59.6	< 0.001
Average well-being (household)	32.1	30.5–33.7	35.0	33.2–36.8	29.8	26.1–33.4	26.2	22.1–30.2	24.6	18.2–30.9	< 0.001
High well-being (household)	35.6	33.4–37.8	42.5	40.0-44.9	23.5	19.8–27.1	29.2	22.6–35.9	28.4	19.3–37.6	< 0.001
Social security	54.0	52.1–55.8	45.3	43.4–47.2	66.9	63.3–70.4	66.7	61.6–71.8	63.1	55.7–70.5	< 0.001
Public clinics per 100,000	13.6	12.3–14.8	9.7	8.8–10.6	21.5	18.0-24.9	17.9	13.9–22.0	11.9	7.2–16.6	< 0.001
Private clinics per 100,000	9.7	9.2–10.3	11.1	10.6–11.7	7.3	6.2–8.4	8.0	6.4–9.6	8.8	5.9–11.7	< 0.001
General practitioners per 1,000	0.6	0.60–0.66	0.6	0.59–0.65	0.7	0.59–0.73	0.7	0.63–0.79	0.5	0.45–0.61	0.71
Medical specialists per 1,000	0.8	0.73–0.88	0.9	0.82–0.99	0.5	0.36–0.59	1.0	0.71–1.27	0.7	0.35–1.03	0.17
Pacific-North	9.9	7.4–12.3	15.8	11.8–19.7	2.0	0.7–3.2	1.0	0.1-7.0	0.2	0.0-1.7	< 0.001
Border	12.7	9.5–15.9	19.7	14.6–24.7	4.8	2.3–7.2	0	0.0–0.0	0	0.0–0.0	< 0.001
Pacific-Central	12.2	8.9–15.5	13.6	9.1–18.0	12.7	5.3–20.1	10.3	1.6–19.0	0	0.0–0.0	0.07
North-Central	12.8	10.2–15.3	18.6	14.6–22.7	6.1	3.9–8.3	2.8	1.0-6.9	1.3	0.0-9.1	< 0.001
Central	9.4	6.6–12.1	6.0	3.9–8.2	18.4	9.4–27.4	8	1.5–14.4	9.5	1.8–17.1	0.05
Cd./State of Mexico	20.6	17.2–24.1	10.4	6.7–14.0	22.3	14.9–29.8	45.8	34.1–57.4	65.4	46.8–84.1	< 0.001
South Pacific	12.7	10.0-15.5	7.2	5.0-9.4	20.2	14.2–26.2	23.9	12.4–35.5	15.1	3.4–47.3	< 0.01
Peninsula	9.8	7.5–12.1	8.7	6.5–11.0	13.5	6.3–20.8	8.2	3.3–13.2	8.5	3.0-21.4	0.77

**Notes**: Percentages reported unless otherwise noted (mean). Standard Errors and *95% Confidence Intervals* (95% CI) are design-based, computed by Taylor series linearization accounting for stratification, clustering of PSUs, and sampling weights. *P for trend* is from survey-weighted regression with deprivation coded as an ordinal score (1–4), using a two-sided Wald test of the linear term. *Estimate* denotes a mean for continuous variables and a percent for categorical variables. *Expanded N* reports population totals (millions) extrapolated with sampling weights. Area type and region follow ENSANUT classifications (urban, rural, metropolitan; macro-regions). *Vulnerable Househ*old is the Presence of household members < 5 y > 65years. *Household well-being categories* (low, average, high) were derived from a principal component index of housing characteristics, services, and assets. *Social security* reflects any reported affiliation (e.g., IMSS, ISSSTE, Pemex, SEDENA, other). *Clinic and physician rates* are facilities per 100,000 and 1,000 respectively population using 2020 municipal denominators. *ENSANUT regions*: Pacific-North (Baja California, Baja California Sur, Nayarit, Sinaloa, Sonora); Border (Chihuahua, Coahuila, Nuevo León, Tamaulipas); Pacific-Center (Colima, Jalisco, Michoacán); Center-North (Aguascalientes, Durango, Guanajuato, Querétaro, San Luis Potosí, Zacatecas); Center (Hidalgo, Tlaxcala, Veracruz); Mexico City and State of Mexico; Pacific-South (Chiapas, Guerrero, Oaxaca); Peninsula (Campeche, Quintana Roo, Yucatán)

## Adjusted gradients across the cascade of care

Adjusted estimates are based on linear regression models controlling for age, sex, socioeconomic and household factors, region, and survey year. Table 3 summarizes adjusted outcome levels by deprivation stratum, and the full set of coefficients is reported in Supplementary Table S3.

**Table 3:** Adjusted prevalence of cardiometabolic outcomes across municipal deprivation strata (DISLI)

	Density-Independent Social Lag Index (DISLI) strata							
	Lowest		Lower-middle		Upper-middle		Highest	
	Mean or Percent	IC95%	Mean or Percent	IC95%	Mean or Percent	IC95%	Mean or Percent	IC95%
Detection of T2D	18.2	16.7–19.7	15.2	13.2–19.7	15.6	12.1–19.1	17.7	14.4–20.1
T2D (self-reported)	11.5	10.7–12.4	11.5	10.4–12.6	10.0	8.5–11.4	9.6	8.2–11.0
Prediabetes (Hba1c-defined)	17.6	15.8–19.4	18.1	15.4–20.8	22.3	16.3–28.2	22.5	16.8–28.2
T2D (Hba1c-defined)	4.46	3.4–5.5	4.68	3.3–6.0	6.05	1.9–10.2	4.2	1.5–7.0
Mean Hba1c (T2D)	8.4	8.1–8.6	8.3	7.9–8.8	8.5	7.8–9.3	9.8	8.3–11.2
Mean glucose value (T2D)	171.9	158.8–184.9	191.3	173.5–209.1	175.3	143.7–206.8	197.0	135.8–258.1
Controlled T2D (HbA1c, %)	31.8	26.2–37.4	36.8	28.0–45.6	27.2	13.3–41.1	13.7	2.6–30.0
Mean glucose value (uncontrolled T2D)	203.7	187.4–220.0	229.8	208.1–251.4	201.2	171.0–231.4	225.1	152.6–297.5
Hypertension (self-reported)	18.2	17.2–19.2	17.3	15.5–19.1	15.7	13.1–18.4	17.5	14.7–20.2
Controlled hypertension	37.6	35.0–40.2	35.1	30.5–39.6	39.6	30.1–49.2	39.4	31.2–47.7
Overweight + obesity	76.4	75.5–77.3	74.4	73.0–75.8	73.1	71.4–74.8	72.8	70.3–75.3
Obesity	40.0	39.0–41.0	36.6	35.4–37.9	35.3	33.1–37.5	36.4	33.2–39.6
Abdominal obesity	82.3	81.4–83.3	79.8	78.3–81.3	80.4	77.9–82.8	80.9	78.1–83.7
Metabolic syndrome	50.8	48.5–53.1	49.2	46.2–52.3	51.9	46.5–57.3	37.2	30.0–44.4
Hypertriglyceridemia	40.4	38.1–42.7	46.0	42.4–49.7	45.3	36.6–54.0	43.2	36.7–49.7
Low HDL‑C	66.7	64.0–69.5	62.9	59.2–66.5	62.4	55.1–69.6	59.9	50.1–69.8
High LDL cholesterol	47.7	44.6–50.8	46.0	42.0–50.0	52.2	46.4–58.1	40.8	29.5–52.0
Total cholesterol	16.6	14.6–18.7	17	13.9–20.0	22.8	16.9–28.8	14.2	8.8–19.7
Impaired renal function	2.1	1.5–2.8	2.8	1.2–4.3	4.7	1.0–8.5	3.9	1.3–6.6

**Notes**: *Estimate* denotes a mean for continuous variables and a percent for categorical variables. *Expanded N* reports population totals (millions) extrapolated with sampling weights. Estimates derived from survey-weighted linear regression models of the outcomes on deprivation, controlling for age, sex, socioeconomic status, education, employment, household vulnerability, social security coverage, region, and survey year. Sampling weights applied for national representativeness. *T2D (self-reported)* refers to a self-reported physician diagnosis. *Prediabetes by HbA1c* is defined as 5.7–6.4%. *T2D by HbA1c* is defined as ≥ 6.5%. *Mean HbA1c (T2D)* and *Mean glucose value (T2D)* denote mean biomarker values among adults with T2D. *Controlled T2D (HbA1c*,* %)* is defined as HbA1c < 7.0% among adults with T2D. *Mean glucose value (uncontrolled T2D)* refers to average glucose levels among adults with uncontrolled T2D. *Hypertension (self-reported)* refers to self-reported physician diagnosis. *Controlled hypertension* is defined as SBP < 130 mmHg and DBP < 80 mmHg among adults with hypertension. *Overweight + obesity* is defined as BMI ≥ 25.0; *Obesity* as BMI ≥ 30.0; and *Abdominal obesity* as waist ≥ 90 cm in men or ≥ 80 cm in women. *Metabolic syndrome* follows *IDF* criteria (central obesity plus ≥ 2 of: triglycerides ≥ 150 mg/dL; HDL-C < 40 mg/dL in men or < 50 mg/dL in women; elevated BP; fasting glucose ≥ 100 mg/dL). *Hypertriglyceridemia* is defined as triglycerides ≥ 150 mg/dL or lipid-lowering therapy. *Low HDL-C* is defined as < 40 mg/dL in men or < 50 mg/dL in women. *High LDL cholesterol* is defined as ≥ 100 mg/dL. *Total cholesterol* is defined as ≥ 200 mg/dL. *Impaired renal function* is defined as CKD-EPI eGFR < 45 mL/min/1.73 m² (stages G3b–G5)

### Screening and detection

The adjusted proportion of adults reporting diabetes screening in the last 12 months showed no evidence of systematic variation across municipal deprivation strata (Table 3; Supplementary Table S3).

### Diagnosis (total prevalence)

For diabetes, adjusted self-reported T2D prevalence was similar between the least and most deprived municipalities (11.5% vs. 9.6%), and the adjusted prevalence of undiagnosed diabetes (HbA1c- defined) remained within a narrow 4–6% band across deprivation strata. Adjusted pre-diabetes prevalence was higher in high deprivation municipalities (22.5% vs. 17.6% in low deprivation), but confidence intervals overlapped (Table 3).

For blood pressure, adjusted self-reported hypertension clustered tightly around 17–18% in every stratum. For lipid outcomes, adjusted levels of HDL-cholesterol, hypertriglyceridemia, elevated LDL-cholesterol, and total cholesterol showed no monotonic pattern across deprivation strata (Supplementary Table S3).

### Awareness

Self-reported awareness measures for T2D, hypertension, and dyslipidemia did not show consistent adjusted gradients across municipal deprivation strata (Table 3; Supplementary Table S3).

### Disease control

Markers of diabetes management showed the largest adjusted differences by deprivation. adjusted share with controlled T2D (HbA1c < 7%) declined from 31.8% in the least deprived stratum to 13.7% in the most deprived. Adjusted mean HbA1c among adults with T2D was higher in the most deprived areas (9.8%) than in the least deprived (8.4%), although estimares were imprecise (Table 3).

For hypertension, the adjusted proportion of treated hypertensives with blood pressure in target range remained near one third across all strata, with no evidence of a deprivation gradient (Table 3; Supplementary Table S3).

### Other cardiometabolic and renal outcomes

Anthropometric indicators displayed an inverse pattern across deprivation strata that remained after adjustment. Obesity prevalence declined from 40.0% in the least deprived to 36.4% in the most deprived municipalities, and overweight + obesity decreased from 76.4% to 72.8%. Abdominal obesity remained close to 80% in all strata. Adjusted metabolic syndrome prevalence was similar across the lower three strata and lowest in the highest deprivation stratum (37.2%), with overlapping confidence intervals (Table 3).

Adjusted point estimates of mild-to-moderate impaired kidney function increased from 2.1% in the least-deprived to 3.9% in the most deprived municipalities, with overlapping confidence intervals (Table 3).

After multivariable adjustment, most associations between municipal deprivation and cardiometabolic outcomes were weak or absent; the main remaining patterns were lower diabetes control in more deprived municipalities and a modest inverse gradient in adiposity and metabolic syndrome, although estimates for these outcomes were imprecise.

## Discussion

Using a density-independent municipal deprivation index, we situated metabolic risk and care performance along the detection–treatment–control cascade. Our analysis revels two distinct gradients: a “control gradient” where municipal deprivation severely hampers the management of diagnosed T2D, and an “inverse risk gradient” where adiposity and metabolic syndrome remain concentrated in less deprived, more urbanized municipalities. These patterns imply that municipal conditions exert differentiated effects across the continuum of care, underscoring the analytic value of distinguishing detection, management, and prevention when assessing inequities.

Our most salient finding is the dissociation between diagnosis and control for T2D. We observed parity in detection across deprivation strata, yet a steep negative gradient in glycemic control, where adjusted control rates dropped from 31.8% in the least deprived to 13.7% in the most deprived municipalities. This mirrors global evidence from the cascade of care literature in LMIC, which consistently identifies the transition from diagnosis to sustained control as the primary leakage point in health systems [36–38].

The parity in detection likely reflects the success of widespread screening campaigns and the expansive coverage of public health systems in identifying cases even in marginalized areas [39], yet overall detection levels remain suboptimal, with substantial proportions of individuals remaining undiagnosed across all socioeconomic strata [39]. However, the failure to translate diagnosis into control in deprived municipalities points to supply-side structural determinants. Deprived municipalities often face interruptions in the continuity of care, characterized by medication stock-outs, limited laboratory capacity for HbA1c monitoring, and lower density of specialized personnel [40, 41]. This pattern suggests that while the Mexican health system has succeeded in finding patients in poor areas, it struggles to retain and treat them effectively, a phenomenon consistent with the “Inverse care law”, where the quality of medical care varies inversely with the need for it [42, 43]. Consequently, addressing these structural inequities requires a policy framework of proportionate universalism, where resource allocation is calibrated to the intensity of local deprivation rather than population size alone [17, 24].

In contrast to the direct gradient observed for glycemic control, we observe an inverse gradient for adiposity, where obesity and metabolic syndrome were more prevalent in wealthier municipalities. This finding is consistent with the “obesity transition” model, which posits that in middle-income countries, obesity burdens shift from the wealthy to the poor in stages [44, 45]. Empirical analyses in Latin America confirm this temporal shift is well underway, with the burden moving toward lower socioeconomic groups as countries develop [4, 46, 47]. However, our results suggest that at the *municipal* level, the obesogenic environment, characterized by higher density of processed food outlets, sedentary service-sector employment, and motorized transport is still more intense in more developed, urbanized municipalities [45]. While individual-level poverty is associated with obesity within the cities, municipal-level development brings environmental risks that raise the baseline BMI for all residents, overriding the protective effect of higher municipal SES [48].

Distinct from both the control gradient in diabetes and the inverse risk gradient in obesity, hypertension and dyslipidemia exhibited high prevalence and low control across strata, with no clear deprivation gradient. This aligns with reviews showing regional heterogeneity in hypertension gradients and widespread under-diagnosis of dyslipidemia across LMICs [36, 39]. The uniformity of poor outcomes suggests these are systemic national failures rather than localized issues of deprivation. The disconnect between widespread screening and effective blood pressure control (stalled at 35–40% regardless of deprivation) highlights a national quality gap that universal coverage alone has not solved.

Looking forward, the next steps are analytic rather than prescriptive. Administrative or panel data in Mexico could be used to test whether post-diagnosis process measures (visit spacing, medication intensification, refill continuity, stock-outs, and routine A1c/lipids/eGFR monitoring) are systematically related to municipal deprivation [36, 49]. In parallel, Mexico has built public–private data partnerships that enable a comprehensive view of the diabetes cascade: the Observatorio Mexicano de Enfermedades No Transmisibles (OMENT) developed with the Secretaría de Salud, academia, and social/ private actors, was created to monitor the national strategy against obesity and diabetes [50, 51]. Fundación Carlos Slim, in collaboration with the Secretaría de Salud, developed dashboards and quality indices now embedded in the national information system (SINBA), including the Tablero de Control de Enfermedades Crónicas, the Sistema de Información en Enfermedades Crónicas (SIC), and the Índices de Calidad de la Atención para diabetes (ICAD) and hypertension (ICAHi), which track retention, consultation effectiveness, and control at the unit, jurisdiction, and state levels with regular updates [52].Small-area rules could be evaluated for their ability to improve diabetes management without widening detection gaps, while obesity prevention and lipid management may be best conceptualized as municipal-level strategies augmented by local calibration regardless of deprivation levels [53–56]. Finally, the municipal governance literature—while largely outside Mexico—offers hypotheses about institutional arrangements that may support equity-oriented chronic disease management; assessing their feasibility in Mexican municipalities is a separate research agenda [57–59].

### Limitations

Several limitations should be noted. First, the cross-sectional design prevents causal inference and cannot rule selective migration (e.g. individuals with poor health remaining in deprived areas). Second, while the DISLI index corrects for population density, assigning individuals to a municipallevel index may result in measurement error regarding their immediate neighborhood environment; high-deprivation pockets exist within affluent municipalities. Third, we relied on single-point biomarkers for control and self-reported diagnosis, which may be subject to recall bias. Finally, we lacked data on specific structural mechanisms such as medication adherence or facility-level quality, which are likely mediators of the observed control gaps.

## Conclusion

Our analysis of the metabolic care cascade in Mexico reveals that municipal deprivation acts as a selective barrier, as it has little impact on whether a patient is diagnosed with T2D, but it is a strong determinant of whether they achieve control. This suggests that the primary driver of metabolic health inequity in Mexico is no longer access to screening, but the quality and continuity of post-diagnosis care in deprived territories.

Therefore, policies should follow the principle of proportionate universalism. While chronic disease services must remain universal, “one-size-fits-all” resource allocation amplifies inequities because deprived municipalities face greater structural barriers to effective treatment. To close the control gap, health policy should allocate proportionally greater resources–specifically for medication supply, HbA1c monitoring, and retention strategies–to municipalities with the highest deprivation levels. Conversely, the concentration of obesity and metabolic syndrome in wealthier, more urbanized municipalities challenges the narrative of deprivation as a risk factor for onset. Prevention strategies regarding food environments and urban planning must be implemented as broad, municipal-level interventions rather than targeted solely at the poorest sectors. Ultimately, reducing metabolic inequities requires a dual approach: strengthening the quality of local health systems in deprived areas to improve control, while regulating the obesogenic environments that drive disease onset in developing urban centers.

## Supplementary Information

Below is the link to the electronic supplementary material.

Supplementary Material 1

## Data Availability

No datasets were generated or analysed during the current study.
