# Supplementary material for: Municipal deprivation and cardiometabolic outcomes in Mexican adults: findings from ENSANUT 2021–2023
Source: Int J Equity Health. 2026 Jan 10;25:19. doi: 10.1186/s12939-025-02754-2 (PMC12828946; doi:10.1186/s12939-025-02754-2)
Supplement: Supplementary file 1 — Supplementary Material 1 [file 12939_2025_2754_MOESM1_ESM.docx]

**Additional file 1. Supplementary methods and tables.**

**Supplementary Methods S1. Construction of the Density-Independent Social Lag Index (DISLI)**

In the analysis of health inequalities in Mexico, the use of socioeconomic status indicators at the ecological (municipal) level presents crucial methodological challenges when comparing urban and rural areas. The Social Lag Index (SLI, or Índice de Rezago Social in Spanish) is a robust and widely used measure for identifying social deprivations; however, previous studies identified a strong, positive, and significant linear correlation between the SLI and population density $(\rho\approx0.73, p-value < 0.001)$. This relationship implies that the traditional SLI tends to systematically penalize dispersed rural zones, automatically assigning them higher lag levels, while favoring dense urban zones, potentially masking pockets of urban poverty. This hinders the analytical distinction between structural material deprivation (poverty) and characteristics inherent to urbanization or geographic dispersion. Without this correction, the standard index conflates the resource constraints of poverty with the logistical isolation of rurality or the infrastructure of urban density, obscuring whether poor metabolic outcomes are driven by socioeconomic disadvantage itself or by the specific environmental context of where people live.

To mitigate this systematic bias and avoid confounding population density with structural poverty in the metabolic care cascade models, we constructed the Density-Independent Social Lag Index (DISLI). The DISLI is operationally defined as the component of variation in social lag that is not linearly explained by the municipality's population density. By isolating this residual component, the DISLI allows for the identification of municipalities that present deprivation conditions worse (or better) than statistically expected given their specific level of urban agglomeration, offering an inequality metric "decoupled" from the rural-urban gradient.

**Data sources and units of analysis**

The index was constructed at the municipal level for all municipalities in Mexico, using information from the year 2020 to temporally align with the data collection period of the ENSANUT 2021-2023 surveys. The following official public access sources were used:

1. **Social lag:** Social Lag Index (SLI) 2020 at the municipal level, calculated and published by the National Council for the Evaluation of Social Development Policy (CONEVAL). This index is the official standard for multidimensional poverty measurement at the territorial level in Mexico.
2. **Population and territory:** 2020 Population and Housing Census from the National Institute of Statistics and Geography (INEGI) and the 2020 National Geostatistical Framework to obtain the precise territorial surface area of each demarcation.

Using unique and standardized municipal codes (concatenation of state identifier 1-32 and a consecutive number for each municipality), we linked the CONEVAL database with INEGI census and geographic data. Municipalities of recent creation or with incomplete information that did not allow for reliable density calculation or linkage with the SLI were excluded from the final analysis. The resulting DISLI, calculated with 2020 data, was subsequently assigned to each individual participant of the ENSANUT 2021, 2022, and 2023 waves, based on their reported municipality of residence at the time of the survey.

**Construction of the standard Social Lag Index (SLI)**

The CONEVAL SLI is a continuous index obtained through the statistical technique of Principal Component Analysis (PCA). This methodology synthesizes information from multiple correlated variables into a single numerical dimension that captures the maximum possible variance. The SLI summarizes ten census indicators of social deprivation, conceptually grouped into four fundamental dimensions of well-being:

- **Education:** Percentage of illiterate population and percentage of population aged 6 to 14 not attending school.
- **Health access:** Percentage of population without entitlement to health services.
- **Housing:** Quality and spaces of the dwelling (dirt floors, flimsy roof material, flimsy wall material, overcrowding).
- **Basic services and assets:** Access to basic services in the dwelling (piped water, drainage, electricity) and availability of basic appliances (washing machine, refrigerator).

The original index possesses standardized continuous values, where a higher numerical value indicates a greater degree of accumulated social deprivation (greater relative lag), while lower values indicate better living conditions.

**Calculation of population density**

In accordance with the standard methodology proposed by INEGI, gross population density was calculated for each municipality $m$ as the arithmetic quotient between the total population recorded in the 2020 Census and the total territorial surface area of the municipality in square kilometers:

$$Density_{m}= {Total population_{m}}/{Area_{i} (km^{2})}$$

This variable captures the physical agglomeration of the population and serves as a continuous and objective proxy for the rural-urban gradient in the adjustment model. Unlike dichotomous classifications (rural/urban), density allows urbanization to be modeled as a continuous spectrum, capturing the heterogeneity between dispersed rural zones, semi-urban localities, and large metropolises.

**Regression model to obtain DISLI**

To statistically decouple social lag from population density, we fitted a simple linear regression model using via Ordinary Least Squares (OLS). In this specification, the municipal SLI functioned as the dependent variable and population density (log) as the independent explanatory variable:

$$SLI_{m}= \beta_{0} + \beta_{1}\cdot log(Density_{m}) + \epsilon_{m}$$

Where:

- $SLI_{m}$ is the Social Lag Index observed in municipality $m$.
- $\beta_{0}$ is the model intercept, representing the base value of social lag when density is theoretically zero.
- $\beta_{1}$ is the regression coefficient capturing the magnitude and direction of the structural association between density and social lag (the average effect of urbanization/dispersion on poverty).
- Log is the natural logarithm, only defined for strictly positive values.
- $\epsilon_{m}$ is the error term or residual for municipality $m$, representing the variance not explained by the model.

The **DISLI** is operationally defined as the value of the standardized residuals  $\hat{\epsilon_{m}}$ derived from this fitted model:

$\epsilon_{m}=SLI_{m}-(\hat{\beta_{0}}-\hat{\beta_{1}}\cdot log(Density_{m})$

By using the residuals, we effectively subtract the portion of the variance in social lag that is linearly associated with population density, leaving only the variation attributable to other structural inequality factors.

Interpretation of these residuals in terms of sign is as follows: Positive values indicate that the municipality has a higher level of social lag than the model would predict based solely on its population density. That is, the municipality presents a structural disadvantage greater than expected, even after considering its level of urbanization or rurality. In turn, negative values indicate that the municipality has a lower level of social lag (i.e. better well-being conditions) than expected; these municipalities have a social performance "better than expected" given their demographic characteristics.

**Categorization of DISLI**

For the main analyses, the continuous DISLI was transformed into an ordinal categorical variable with four levels (contrary to the five levels reported by CONEVAL). To perform this categorization, the Dalenius & Hodges optimal stratification algorithm (cumulative square root frequency method) was used. This method is superior to simple quartile division because it seeks to minimize internal variance within each stratum and maximize variance between strata, creating groups that are more homogeneous and representative of distinct deprivation levels.

Prior to applying the algorithm, an auxiliary positive linear transformation $(DISLI + |min(DISLI)| +\delta)$ was performed to ensure all values were positive, complying with the mathematical requirements of the Dalenius & Hodges method without altering the relative distribution or the order of municipalities.

The resulting cut-off points allowed municipalities to be classified into four levels of density-independent deprivation: 1) Low (municipalities with the lowest deprivation relative to their density, i.e. best conditions; 2) Medium-Low (municipalities with intermediate conditions, tending towards favorable); 3) Medium-High **(**municipalities with intermediate conditions, tending towards unfavorable); and 4) High (municipalities with the highest deprivation relative to their density, i.e. worst structural conditions).

**Computational implementation**

The entire workflow, from data processing and cleaning to model fitting and final stratification, was performed using the R statistical programming language. Standard and specialized libraries were employed to ensure reproducibility:

- Base R functions (lm) were used for fitting the linear regression model.
- The *stratification* package (specifically the strata.cumrootf function) was used for the implementation of the Dalenius & Hodges algorithm.

**Table S1 State composition of municipal stratum shifts (DISLI vs original Social Lag Index)**

|  | Shift in deprivation stratum (DISLI − original): column percentages (each shift category sums to 100%) | | | | | |
| --- | --- | --- | --- | --- | --- | --- |
| State | ↓2 | ↓1 | No change | ↑1 | ↑2 | ↑3 |
| Aguascalientes | 0 | 0 | 2 | 0 | 0 | 0 |
| Baja California | 0 | 0 | 2 | 0 | 0 | 0 |
| Baja California Sur | 0 | 0 | 1 | 0 | 0 | 0 |
| Campeche | 0 | 0 | 2 | 0 | 0 | 0 |
| Coahuila | 0 | 1 | 3 | 0 | 0 | 0 |
| Colima | 0 | 0 | 2 | 3 | 0 | 0 |
| Chiapas | 14 | 10 | 3 | 0 | 0 | 0 |
| Chihuahua | 0 | 1 | 4 | 0 | 0 | 0 |
| Ciudad de México | 0 | 0 | 1 | 11 | 56 | 50 |
| Durango | 0 | 1 | 3 | 0 | 0 | 0 |
| Guanajuato | 0 | 9 | 8 | 0 | 0 | 0 |
| Guerrero | 29 | 7 | 2 | 0 | 0 | 0 |
| Hidalgo | 0 | 7 | 4 | 0 | 0 | 0 |
| Jalisco | 0 | 1 | 3 | 6 | 11 | 0 |
| Estado de México | 14 | 5 | 7 | 23 | 33 | 33 |
| Michoacán | 0 | 3 | 4 | 3 | 0 | 0 |
| Morelos | 0 | 2 | 4 | 6 | 0 | 0 |
| Nayarit | 0 | 2 | 3 | 0 | 0 | 0 |
| Nuevo León | 0 | 1 | 4 | 6 | 0 | 0 |
| Oaxaca | 0 | 6 | 3 | 11 | 0 | 0 |
| Puebla | 29 | 10 | 3 | 6 | 0 | 0 |
| Querétaro | 0 | 2 | 2 | 0 | 0 | 0 |
| Quintana Roo | 14 | 1 | 2 | 0 | 0 | 0 |
| San Luis Potosí | 0 | 4 | 2 | 0 | 0 | 0 |
| Sinaloa | 0 | 2 | 3 | 0 | 0 | 0 |
| Sonora | 0 | 0 | 4 | 0 | 0 | 0 |
| Tabasco | 0 | 6 | 1 | 0 | 0 | 0 |
| Tamaulipas | 0 | 1 | 3 | 3 | 0 | 0 |
| Tlaxcala | 0 | 1 | 6 | 14 | 0 | 17 |
| Veracruz | 0 | 5 | 4 | 9 | 0 | 0 |
| Yucatán | 0 | 7 | 2 | 0 | 0 | 0 |
| Zacatecas | 0 | 5 | 3 | 0 | 0 | 0 |
| Note: Unit of analysis is the municipality; results are based on the municipality-level classification after collapsing to one record per CVE_MUN; original municipal deprivation strata are derived from the 2020 Social Lag Index (IMN-2020) and DISLI strata are derived from the density-independent social-lag index; to ensure comparability, original IMN strata were re-coded so that higher categories reflect higher deprivation (Low, Middle, High, Very high), matching the DISLI ordering; shift is defined as DISLI stratum minus original stratum (re-coded); negative values indicate reclassification to a less deprived category under DISLI and positive values indicate reclassification to a more deprived category; arrows denote the number of strata moved (e.g., ↓2 indicates a move of two categories toward lower deprivation and ↑3 indicates a move of three categories toward higher deprivation); entries are column percentages so each shift column sums to 100% and shows the share of municipalities within a given shift category that are located in each state; some state-by-shift cells are based on a small number of municipalities and should be interpreted with caution. | | | | | | |

**Table S2 Unadjusted prevalence and means of cardiometabolic risk factors among Mexican adults, by municipality-level deprivation (DISLI), ENSANUT 2021-2023**

|  | **Density-Independent Social Lag Index (DISLI) strata** | | | | | | | | | | |
| --- | --- | --- | --- | --- | --- | --- | --- | --- | --- | --- | --- |
|  | **Total** |  | **Lowest** |  | **Lower-middle** |  | **Upper-middle** |  | **Highest** |  |  |
| Sample Size (N) | 32,087 |  | 20,647 |  | 7,285 |  | 2,641 |  | 1,514 |  |  |
| Expanded N (Millions) | *85.4* |  | *50.2* |  | *19.7* |  | *10.4* |  | *5.1* |  |  |
|  | **Estimate** | **CI 95%**† | **Estimate** | **CI 95%** | **Estimate** | **CI 95%** | **Estimate** | **CI 95%** | **Estimate** | **CI 95%** | **P Trend** |
| **Type 2 Diabetes (T2D)** | | | | | | | | | | | |
| **Detection of T2D** | 15.7 | 14.6-16.8 | 16.4 | 15.0-17.9 | 14.4 | 11.7-17.0 | 14.6 | 11.9-17.4 | 15.6 | 13.0-18.2 | 0.28 |
| **Prediabetes (Hba1c-defined)** | 18.7 | 17.0-20.3 | 17.7 | 15.7-19.8 | 18.2 | 15.1-21.2 | 22.5 | 16.2-28.8 | 22.2 | 15.3-29.1 | 0.08 |
| **T2D (Hba1c-defined)** | 4.6 | 3.7-5.5 | 4.5 | 3.4-5.6 | 4.5 | 3.2-5.9 | 5.8 | 1.7-9.9 | 4 | 1.4-6.6 | 0.74 |
| **T2D (self-reported)** | 10.7 | 10.1-11.2 | 10.7 | 10.0-11.5 | 11.3 | 10.3-12.3 | 9.9 | 8.1-11.7 | 9.6 | 7.8-11.3 | 0.3 |
| **Mean Hba1c (T2D)** | 8.4 | 8.1-8.7 | 8.3 | 8.0-8.6 | 8.3 | 7.8-8.9 | 8.8 | 7.5-10.1 | 9.7 | 7.8-11.5 | 0.2 |
| **Mean glucose value (T2D)** | 175.6 | 164.4-186.7 | 169.6 | 155.4-183.9 | 189.6 | 168.8-210.4 | 176.3 | 140.8-211.9 | 192 | 114.5-269.4 | 0.28 |
| **Controlled T2D (HbA1c, %)** | 31.6 | 26.3-36.9 | 30.4 | 23.5-37.4 | 37.7 | 27.9-47.6 | 28.9 | 12.0-45.8 | 12.8 | 3.8-35.4 | 0.81 |
| **Mean glucose value (uncontrolled T2D)** | 206.4 | 190.8-222.1 | 198.4 | 177.9-218.9 | 229.7 | 202.7-256.6 | 205.7 | 169.3-242.2 | 216.4 | 117.0-315.9 | 0.33 |
| **Hypertension** |  |  |  |  |  |  |  |  |  |  |  |
| **Hypertension (self-reported)** | 16.3 | 15.5-17.2 | 16.8 | 15.6-17.9 | 16.0 | 14.6-17.5 | 14.5 | 11.9-17.2 | 16.6 | 13.6-19.7 | 0.26 |
| **Elevated blood pressure** | 33.9 | 32.7-35.2 | 35.2 | 33.5-37.0 | 34.1 | 31.9-36.3 | 30.5 | 27.5-33.5 | 26.9 | 22.5-31.3 | <0.001 |
| **Controlled hypertension** | 37.4 | 35.1-39.7 | 37.3 | 34.6-40.0 | 35.4 | 30.4-40.4 | 40.2 | 31.2-49.3 | 40.5 | 28.9-52.0 | 0.54 |
| **Obesity and Metabolic syndrome** | | | | | | | | | | | |
| **Mean BMI** | 29.0 | 28.8-29.1 | 29.3 | 29.1-29.4 | 28.7 | 28.4-29.0 | 28.5 | 28.2-28.8 | 28.4 | 27.8-28.9 | <0.001 |
| **Overweight +**  **obesity** | 73.8 | 72.7-74.8 | 74.7 | 73.4-76.0 | 73.1 | 70.8-75.4 | 71.4 | 68.8-74.1 | 71.9 | 68.2-75.5 | 0.02 |
| **Obesity** | 38.3 | 37.1-39.6 | 40 | 38.3-41.7 | 36.5 | 34.5-38.5 | 34.7 | 31.5-38.0 | 36.4 | 30.3-42.5 | 0.01 |
| **Abdominal obesity** | 81.4 | 80.4-82.3 | 82.3 | 81.1-83.5 | 79.9 | 77.8-81.9 | 80.1 | 77.4-82.8 | 80.7 | 77.3-84.1 | 0.08 |
| **Metabolic syndrome** | 49.2 | 47.1-51.2 | 50 | 47.3-52.7 | 48.9 | 45.2-52.7 | 52.4 | 46.6-58.1 | 37 | 29.3-44.6 | 0.07 |
| **Lipid disorders** | | | | | | | | | | | |
| **Hypertriglyceridemia** | 42.8 | 40.7-44.8 | 40.8 | 38.2-43.4 | 45.9 | 42.0-49.9 | 46.1 | 38.0-54.3 | 43.5 | 38.5-48.6 | 0.08 |
| **Low HDL‑C** | 64.8 | 62.4-67.3 | 66.1 | 62.9-69.3 | 62.9 | 58.9-67.0 | 64.4 | 56.3-72.5 | 60.1 | 46.7-73.6 | 0.29 |
| **High LDL cholesterol** | 47.5 | 44.8-50.1 | 48.2 | 44.6-51.8 | 45.5 | 40.9-50.0 | 51.5 | 44.4-58.7 | 40.6 | 26.7-54.4 | 0.57 |
| **Total cholesterol** | 17.4 | 15.5-19.4 | 17.1 | 14.5-19.8 | 16.7 | 13.7-19.6 | 22.1 | 16.5-27.7 | 14.8 | 6.2-23.3 | 0.68 |
| **Kidney disease** | | | | | | | | | | | |
| **Impaired renal function** | 2.9 | 2.1-3.6 | 2.3 | 1.6-3.0 | 3 | 1.5-4.5 | 4.9 | 2.3-10.0 | 4.9 | 1.9-12.2 | 0.07 |

**Notes:** IDF refers to the International Diabetes Federation; T2D to Type 2 Diabetes; HbA1c to Hemoglobin A1c; CKD-EPI to the Chronic Kidney Disease Epidemiology Collaboration equation; SBP to Systolic Blood Pressure; DBP to Diastolic Blood Pressure; and BMI to Body Mass Index (weight/height², kg/m²). Standard Errors and 95% Confidence Intervals (95% CI) are design-based, computed by Taylor series linearization accounting for stratification, clustering of PSUs, and sampling weights. P for trend is from survey-weighted regression with deprivation coded as an ordinal score (1–4), using a two-sided Wald test of the linear term. Estimate denotes a mean for continuous variables and a percent for categorical variables. Expanded N reports population totals (millions) extrapolated with sampling weights. Detection of T2D refers to whether the individual underwent testing or screening for diabetes in the last 12 months. Hyperglycemia is defined as plasma glucose ≥100 mg/dL or prior T2D diagnosis. Prediabetes by HbA1c is defined as 5.7–6.4%. T2D by HbA1c is defined as ≥6.5%. HbA1c value with T2D and Glucose value with T2D denote mean biomarker values among adults with diabetes. Control of T2D by HbA1c is defined as HbA1c <7.0% among adults with diabetes. Elevated blood pressure is defined as SBP ≥130 mmHg or DBP ≥80 mmHg (2017 ACC/AHA). Controlled hypertension is defined as SBP <130 and DBP <80 mmHg among adults with hypertension. BMI is calculated as weight/height² (kg/m²). Overweight + obesity is defined as BMI ≥25.0; Obesity as BMI ≥30.0; and Abdominal obesity as waist ≥90 cm in men or ≥80 cm in women. Metabolic syndrome follows the IDF criteria (central obesity plus ≥2 of: triglycerides ≥150 mg/dL or treatment; HDL-C <40 mg/dL in men or <50 mg/dL in women; elevated blood pressure; fasting glucose ≥100 mg/dL). Hypertriglyceridemia is defined as triglycerides ≥150 mg/dL or lipid-lowering therapy. Low HDL-C is defined as <40 mg/dL in men or <50 mg/dL in women. High LDL-C is defined as ≥100 mg/dL. Total cholesterol is defined as ≥200 mg/dL. Impaired renal function is defined as CKD-EPI eGFR <45 mL/min/1.73 m² (stages G3b–G5).

**Table S3 Regression Models output 1/3**

|  | **Biomarkers** | | | | | | | | |
| --- | --- | --- | --- | --- | --- | --- | --- | --- | --- |
|  | **Prediabetes (Hba1c-defined)** | **T2D (Hba1c-defined)** | **Controlled T2D (HbA1c, %)** | **Mean Hba1c (T2D)** | **Impaired renal function** | | **Mean Glucose value (T2D)** | **Mean Glucose value (uncontrolled T2D)** | **Hypertriglyceridemia** |
| **Covariates** | **Coefficient (standard error)** | **Coefficient (standard error)** | **Coefficient (standard error)** | **Coefficient (standard error)** | **Coefficient (standard error)** | | **Coefficient (standard error)** | **Coefficient (standard error)** | **Coefficient (standard error)** |
| **DISLI (Deprivation level)** |  |  |  |  |  | |  |  |  |
| Middle | -0.022 (0.021) | 0.009 (0.011) | 0.095 (0.062) | -0.217 (0.296) | 0.006 (0.010) | 14.682 (11.699) | | 21.115 (14.356) | -0.002 (0.027) |
| High | -0.001 (0.037) | 0.023 (0.022) | -0.028 (0.096) | 0.179 (0.468) | 0.021 (0.020) | -0.796 (20.282) | | -14.393 (22.905) | -0.014 (0.051) |
| Very high | 0.011 (0.035) | 0.003 (0.017) | -0.131 (0.105) | 1.314 (0.796) | 0.020 (0.016) | 46.277 (33.789) | | 49.078 (40.273) | -0.046 (0.042) |
| **Well-being tercile** |  |  |  |  |  |  | |  |  |
| Well-being tercile = 2 | -0.023 (0.020) | 0.002 (0.012) | 0.055 (0.062) | -0.689 (0.267)** | 0.005 (0.009) | -21.814 (11.179) | | -16.378 (14.080) | -0.025 (0.026) |
| Well-being tercile = 3 | -0.001 (0.023) | 0.016 (0.013) | 0.011 (0.064) | -0.874 (0.295)** | 0.003 (0.009) | -27.525 (12.104)* | | -30.483 (14.601)* | -0.019 (0.033) |
| **Age (years)** | 0.007 (0.001)*** | 0.001 (0.000)*** | 0.005 (0.003) | -0.039 (0.015)* | 0.002 (0.000)*** | -2.256 (0.586)*** | | -2.897 (0.599)*** | 0.003 (0.001)*** |
| **Sex** |  |  |  |  |  |  | |  |  |
| Female | 0.033 (0.017) | 0.005 (0.011) | -0.059 (0.059) | 0.352 (0.290) | -0.005 (0.007) | -5.290 (12.813) | | -11.864 (15.816) | -0.091 (0.025)*** |
| **Marital status** |  |  |  |  |  |  | |  |  |
| Cohabiting | -0.012 (0.020) | 0.013 (0.013) | -0.003 (0.071) | 0.403 (0.391) | 0.007 (0.010) | 24.862 (17.349) | | 33.031 (18.593) | -0.029 (0.032) |
| Separated/Divorced/Single | 0.004 (0.020) | 0.008 (0.012) | 0.165 (0.076)* | -0.621 (0.286)* | 0.004 (0.010) | -12.877 (13.650) | | 9.239 (18.793) | -0.031 (0.029) |
| Widowed | 0.028 (0.044) | 0.002 (0.024) | 0.021 (0.069) | 0.071 (0.318) | 0.008 (0.017) | -3.409 (10.177) | | -1.726 (12.563) | -0.009 (0.043) |
| **Body mass index (kg/m²)** | 0.010 (0.001)*** | 0.003 (0.001)** | 0.016 (0.004)*** | -0.088 (0.018)*** | -0.001 (0.001) | -1.692 (0.843)* | | -0.862 (0.993) | 0.017 (0.002)*** |
| **Education** |  |  |  |  |  |  | |  |  |
| Secondary | -0.036 (0.027) | -0.023 (0.013) | 0.006 (0.056) | -0.307 (0.280) | 0.007 (0.010) | -17.706 (11.143) | | -21.466 (13.754) | 0.019 (0.036) |
| High school / Technical | -0.070 (0.027)* | -0.012 (0.018) | 0.070 (0.082) | -0.205 (0.334) | 0.023 (0.011)* | -16.883 (13.262) | | -26.485 (16.672) | -0.012 (0.034) |
| College or Postgraduate | -0.063 (0.031)* | -0.040 (0.014)** | -0.026 (0.080) | 0.264 (0.373) | 0.010 (0.011) | -13.374 (18.260) | | -34.048 (24.037) | -0.010 (0.045) |
| **Smoking status** |  |  |  |  |  |  | |  |  |
| Former smoker | 0.008 (0.023) | 0.002 (0.012) | 0.020 (0.059) | -0.152 (0.268) | 0.018 (0.013) | 5.011 (14.020) | | 12.365 (18.011) | 0.007 (0.036) |
| Current smoker | -0.044 (0.017)** | 0.013 (0.014) | -0.029 (0.074) | 0.406 (0.356) | -0.011 (0.006)* | 16.111 (14.567) | | 8.657 (17.898) | 0.052 (0.037) |
| **Currently Working** |  |  |  |  |  |  | |  |  |
| Yes | 0.017 (0.017) | 0.004 (0.012) | -0.028 (0.055) | 0.122 (0.323) | -0.013 (0.009) | 11.638 (11.805) | | 17.389 (12.857) | -0.002 (0.022) |
| **Vulnerable household** |  |  |  |  |  |  | |  |  |
| Yes | 0.027 (0.023) | -0.033 (0.012)** | 0.091 (0.066) | -0.078 (0.262) | 0.015 (0.010) | -3.524 (12.809) | | 12.552 (15.821) | -0.019 (0.025) |
| **Social security (covered)** |  |  |  |  |  |  | |  |  |
| Yes | -0.012 (0.017) | 0.004 (0.011) | -0.009 (0.051) | -0.130 (0.244) | -0.013 (0.009) | -9.796 (8.785) | | -6.991 (10.586) | -0.016 (0.024) |
| **Region** |  |  |  |  |  |  | |  |  |
| Border | 0.052 (0.030) | 0.005 (0.023) | -0.101 (0.095) | 0.714 (0.400) | 0.010 (0.009) | 39.407 (17.219)* | | 56.541 (18.906)** | 0.077 (0.043) |
| Pacific Center | 0.017 (0.041) | -0.033 (0.020) | -0.196 (0.099)* | 0.438 (0.476) | 0.025 (0.017) | 17.932 (22.270) | | 6.395 (26.839) | 0.069 (0.051) |
| Center North | 0.020 (0.027) | -0.019 (0.018) | -0.009 (0.080) | 0.197 (0.346) | 0.007 (0.008) | 4.596 (13.433) | | -0.985 (17.748) | 0.124 (0.041)** |
| Center | 0.064 (0.032)* | 0.023 (0.022) | -0.200 (0.103) | 0.870 (0.467) | -0.003 (0.012) | 58.425 (22.683)* | | 52.813 (29.737) | 0.126 (0.050)* |
| Mexico City / State of Mexico | 0.098 (0.033)** | -0.023 (0.021) | -0.035 (0.094) | -0.125 (0.391) | 0.011 (0.010) | -9.270 (16.860) | | -15.935 (18.915) | 0.214 (0.047)*** |
| Pacific South | 0.070 (0.032)* | -0.001 (0.021) | -0.125 (0.093) | 0.094 (0.464) | 0.022 (0.013) | 10.793 (22.317) | | 3.446 (26.609) | 0.115 (0.048)* |
| Peninsula | 0.035 (0.034) | -0.022 (0.018) | -0.261 (0.085)** | 1.352 (0.473)** | 0.005 (0.009) | 47.598 (19.310)* | | 33.780 (24.778) | 0.115 (0.044)** |
| **Survey year** |  |  |  |  |  |  | |  |  |
| 2022 | -0.057 (0.019)** | 0.001 (0.012) | 0.106 (0.061) | -0.326 (0.288) | 0.007 (0.008) | -27.980 (16.081) | | -19.917 (19.655) | -0.066 (0.026)* |
| 2023 | -0.056 (0.019)** | 0.010 (0.009) | -0.038 (0.052) | 0.177 (0.270) | 0.010 (0.009) | -16.064 (13.617) | | -27.188 (15.674) | -0.103 (0.024)*** |
| **Health system supply** |  |  |  |  |  |  | |  |  |
| Public clinics rate | 0.088 (0.070) | -0.050 (0.033) | 0.331 (0.183) | -2.396 (0.994)* | 0.012 (0.021) | -78.045 (45.404) | | -44.333 (56.817) | 0.104 (0.095) |
| Private clinics rate | 0.283 (0.180) | -0.079 (0.099) | 1.215 (0.513)* | -8.501 (2.658)** | -0.069 (0.074) | -285.247 (119.189)* | | -252.745 (141.302) | -0.266 (0.237) |
| General practitioners (total) | -0.322 (2.635) | -1.041 (1.386) | -6.298 (6.372) | 36.726 (36.525) | 0.091 (1.205) | 1595.947 (1739.289) | | 136.237 (2339.512) | -2.009 (3.488) |

**Continue 2/3**

|  | **Biomarkers** | | |  | **Clinic characteristics** | | |
| --- | --- | --- | --- | --- | --- | --- | --- |
|  | **Low HDL‑C** | **Hyperglycemia** | **Total cholesterol** | **Detection of T2D** | **T2D (self-reported)** | **Hypertension (self-reported)** | **Detection of T2D** |
| **Covariates** | **Coefficient (standard error)** | **Coefficient (standard error)** | **Coefficient (standard error)** | **Coefficient (standard error)** | **Coefficient (standard error)** | **Coefficient (standard error)** | **Coefficient (standard error)** |
| **DISLI (Deprivation level)** |  |  |  |  |  |  |  |
| Middle | -0.045 (0.029) | 0.009 (0.026) | -0.030 (0.021) | -0.009 (0.015) | 0.002 (0.008) | 0.001 (0.010) | -0.009 (0.015) |
| High | -0.048 (0.048) | 0.015 (0.038) | 0.019 (0.037) | -0.025 (0.023) | -0.013 (0.011) | -0.023 (0.016) | -0.025 (0.023) |
| Very high | -0.085 (0.060) | -0.081 (0.041)* | -0.059 (0.036) | -0.011 (0.022) | -0.014 (0.011) | -0.005 (0.016) | -0.011 (0.022) |
| **Well-being tercile** |  |  |  |  |  |  |  |
| Well-being tercile = 2 | -0.016 (0.024) | 0.019 (0.025) | 0.004 (0.020) | -0.006 (0.013) | 0.010 (0.008) | 0.008 (0.010) | -0.006 (0.013) |
| Well-being tercile = 3 | -0.009 (0.028) | 0.017 (0.029) | 0.007 (0.024) | 0.008 (0.015) | 0.003 (0.009) | -0.001 (0.011) | 0.008 (0.015) |
| **Age (years)** | -0.002 (0.001)** | 0.008 (0.001)*** | 0.005 (0.001)*** | 0.003 (0.000) | 0.005 (0.000)*** | 0.008 (0.000)*** | 0.003 (0.000)*** |
| **Sex** |  |  |  |  |  |  |  |
| Female | 0.206 (0.023)*** | 0.010 (0.023) | -0.034 (0.019) | -0.004 (0.011) | -0.000 (0.007) | -0.000 (0.009) | -0.004 (0.011) |
| **Marital status** |  |  |  |  |  |  |  |
| Cohabiting | -0.011 (0.026) | 0.007 (0.031) | 0.040 (0.021) | -0.035** (0.013) | -0.013 (0.008) | 0.001 (0.010) | -0.035 (0.013)** |
| Separated/Divorced/Single | -0.025 (0.027) | -0.034 (0.026) | -0.010 (0.018) | -0.016 (0.012) | -0.005 (0.007) | 0.014 (0.010) | -0.016 (0.012) |
| Widowed | -0.034 (0.037) | -0.010 (0.035) | 0.008 (0.034) | -0.016 (0.022) | 0.003 (0.016) | 0.026 (0.022) | -0.016 (0.022) |
| **Body mass index (kg/m²)** | 0.014 (0.002)*** | 0.014 (0.002)*** | 0.001 (0.001) | 0.004 (0.001)*** | 0.002 (0.001)*** | 0.009 (0.001)*** | 0.004 (0.001)*** |
| **Education** |  |  |  |  |  |  |  |
| Secondary | -0.047 (0.028) | -0.007 (0.030) | 0.027 (0.022) | 0.044 (0.015)* | -0.008 (0.010) | -0.007 (0.011) | 0.044 (0.015)** |
| High school / Technical | -0.039 (0.038) | -0.028 (0.031) | 0.046 (0.025) | 0.044 (0.016)* | -0.023 (0.011)* | -0.017 (0.014) | 0.044 (0.016)** |
| College or Postgraduate | -0.084 (0.041)* | -0.049 (0.034) | 0.076 (0.028)** | 0.056 (0.019)* | -0.039 (0.013)** | -0.022 (0.016) | 0.056 (0.019)** |
| **Smoking status** |  |  |  |  |  |  |  |
| Former smoker | 0.057 (0.029) | 0.056 (0.030) | -0.021 (0.021) | 0.007 (0.013) | 0.015 (0.009) | 0.003 (0.011) | 0.007 (0.013) |
| Current smoker | 0.033 (0.030) | -0.029 (0.029) | -0.014 (0.022) | 0.003 (0.013) | -0.012 (0.009) | -0.024 (0.010)* | 0.003 (0.013) |
| **Currently Working** |  |  |  |  |  |  |  |
| Yes | 0.012 (0.023) | -0.001 (0.021) | 0.012 (0.018) | 0.004 (0.010) | -0.031 (0.009)*** | -0.056 (0.009)*** | 0.004 (0.010) |
| **Vulnerable household** |  |  |  |  |  |  |  |
| Yes | 0.017 (0.024) | -0.018 (0.025) | -0.001 (0.021) | 0.018 (0.014) | -0.005 (0.007) | 0.007 (0.010) | 0.018 (0.014) |
| **Social security (covered)** |  |  |  |  |  |  |  |
| Yes | -0.009 (0.024) | -0.028 (0.025) | 0.019 (0.016) | -0.087 (0.013)*** | -0.025 (0.007)*** | -0.025 (0.007)*** | -0.087 (0.013)*** |
| **Region** |  |  |  |  |  |  |  |
| Border | 0.000 (0.046) | 0.009 (0.048) | -0.031 (0.034) | -0.004 (0.022) | 0.015 (0.016) | -0.022 (0.015) | -0.004 (0.022) |
| Pacific Center | -0.066 (0.049) | -0.040 (0.049) | -0.056 (0.036) | -0.008 (0.030) | 0.011 (0.017) | -0.056 (0.016)*** | -0.008 (0.030) |
| Center North | -0.001 (0.038) | -0.004 (0.035) | -0.000 (0.031) | 0.025 (0.018) | 0.017 (0.015) | 0.007 (0.014) | 0.025 (0.018) |
| Center | 0.098 (0.044)* | 0.067 (0.046) | -0.026 (0.035) | 0.036 (0.020) | 0.038 (0.017)* | -0.016 (0.022) | 0.036 (0.020) |
| Mexico City / State of Mexico | -0.034 (0.049) | -0.070 (0.045) | 0.055 (0.037) | 0.057 (0.024) | 0.000 (0.017) | -0.007 (0.015) | 0.057 (0.024)* |
| Pacific South | 0.109 (0.041)** | -0.002 (0.043) | -0.065 (0.031)* | 0.013 (0.020) | 0.020 (0.015) | -0.045 (0.015)** | 0.013 (0.020) |
| Peninsula | 0.042 (0.040) | -0.048 (0.041) | -0.023 (0.033) | 0.030 (0.019) | 0.012 (0.015) | -0.036 (0.017)* | 0.030 (0.019) |
| **Survey year** |  |  |  |  |  |  |  |
| 2022 | 0.167 (0.025)*** | -0.022 (0.026) | -0.127 (0.022)*** | 0.039 (0.010)*** | -0.000 (0.007) | -0.002 (0.009) | 0.039 (0.010)*** |
| 2023 | 0.192 (0.028)*** | 0.000 (0.025) | -0.147 (0.022)*** | 0.162 (0.014)*** | -0.002 (0.008) | 0.017 (0.011) | 0.162 (0.014)*** |
| **Health system supply** |  |  |  | 0.101(0.064) |  |  |  |
| Public clinics rate | -0.110 (0.092) | -0.110 (0.080) | 0.083 (0.074) | 0.207(0.133) | -0.051 (0.025)* | 0.063 (0.038) | 0.101 (0.064) |
| Private clinics rate | -0.392 (0.267) | 0.063 (0.211) | -0.128 (0.206) | -0.735(2.185) | -0.068 (0.068) | 0.168 (0.099) | 0.207 (0.133) |
| General practitioners (total) | 2.104 (3.213) | -3.968 (3.021) | -1.436 (2.493) | -0.009 (0.015) | 0.532 (1.002) | -0.928 (1.319) | -0.735 (2.185) |

**Continue 3/3**

|  |  | |  |  |  |  |
| --- | --- | --- | --- | --- | --- | --- |
|  | **Overweight/obesity** | **Obesity** | | **Abdominal obesity** | **Controlled hypertension** | **Metabolic syndrome** |
| **Covariates** | **Coefficient (standard error)** | **Coefficient (standard error)** | | **Coefficient (standard error)** | **Coefficient (standard error)** | **Coefficient (standard error)** |
| **DISLI (Deprivation level)** |  |  | |  |  |  |
| Middle | 0.004 (0.009) | -0.003 (0.009) | | -0.012 (0.010) | -0.026 (0.033) | -0.014 (0.022) |
| High | -0.013 (0.011) | -0.003 (0.013) | | -0.016 (0.016) | -0.005 (0.057) | 0.011 (0.033) |
| Very high | -0.004 (0.015) | 0.024 (0.018) | | 0.004 (0.017) | -0.014 (0.053) | -0.114 (0.043)** |
| **Well-being tercile** |  |  | |  |  |  |
| Well-being tercile = 2 | 0.031 (0.009)** | 0.006 (0.009) | | 0.016 (0.010) | -0.042 (0.031) | -0.026 (0.025) |
| Well-being tercile = 3 | 0.030 (0.011)** | -0.022 (0.011)* | | 0.026 (0.012)* | -0.036 (0.036) | -0.042 (0.025) |
| **Age (years)** | 0.002 (0.000)*** | -0.000 (0.000) | | 0.004 (0.000)*** | -0.003 (0.001)** | 0.008 (0.001)*** |
| **Sex** |  |  | |  |  |  |
| Female | -0.014 (0.009) | 0.017 (0.008)* | | 0.111 (0.009)*** | 0.093 (0.029)** | -0.017 (0.021) |
| **Marital status** |  |  | |  |  |  |
| Cohabiting | -0.003 (0.010) | 0.014 (0.011) | | 0.007 (0.011) | -0.015 (0.038) | -0.080 (0.036)* |
| Separated/Divorced/Single | -0.034 (0.009)*** | 0.009 (0.009) | | -0.059 (0.010)*** | -0.010 (0.034) | -0.014 (0.026) |
| Widowed | -0.024 (0.013) | 0.000 (0.012) | | -0.041 (0.011)*** | -0.030 (0.032) | -0.028 (0.035) |
| **Body mass index (kg/m²)** | 0.049 (0.001)*** | 0.066 (0.001)*** | | 0.035 (0.001)*** | -0.002 (0.003) | 0.030 (0.002)*** |
| **Education** |  |  | |  |  |  |
| Secondary | 0.013 (0.010) | -0.007 (0.011) | | 0.022 (0.011)* | 0.033 (0.032) | 0.036 (0.037) |
| High school / Technical | 0.008 (0.013) | -0.009 (0.013) | | 0.015 (0.011) | 0.102 (0.040)* | -0.007 (0.036) |
| College or Postgraduate | 0.014 (0.014) | -0.021 (0.017) | | 0.021 (0.014) | 0.050 (0.041) | -0.046 (0.039) |
| **Smoking status** |  |  | |  |  |  |
| Former smoker | -0.011 (0.010) | 0.003 (0.009) | | -0.004 (0.011) | -0.010 (0.032) | -0.005 (0.027) |
| Current smoker | -0.014 (0.011) | -0.023 (0.011)* | | -0.006 (0.011) | 0.020 (0.040) | 0.020 (0.035) |
| **Currently Working** |  |  | |  |  |  |
| Yes | 0.017 (0.008)* | 0.007 (0.007) | | 0.027 (0.008)*** | -0.025 (0.025) | 0.017 (0.021) |
| **Vulnerable household** |  |  | |  |  |  |
| Yes | -0.026 (0.010)* | -0.004 (0.010) | | -0.004 (0.010) | 0.063 (0.039) | -0.027 (0.027) |
| **Social security (covered)** |  |  | |  |  |  |
| Yes | 0.002 (0.008) | -0.007 (0.008) | | -0.002 (0.009) | -0.036 (0.028) | -0.005 (0.022) |
| **Region** |  |  | |  |  |  |
| Border | -0.010 (0.011) | 0.014 (0.016) | | 0.003 (0.011) | -0.005 (0.038) | -0.002 (0.033) |
| Pacific Center | 0.014 (0.016) | 0.004 (0.016) | | 0.031 (0.017) | -0.006 (0.058) | -0.041 (0.039) |
| Center North | 0.006 (0.013) | -0.009 (0.014) | | 0.024 (0.013) | 0.048 (0.034) | 0.013 (0.031) |
| Center | -0.017 (0.015) | -0.021 (0.016) | | 0.018 (0.016) | -0.037 (0.051) | 0.038 (0.037) |
| Mexico City / State of Mexico | 0.032 (0.012)** | -0.010 (0.013) | | 0.037 (0.015)* | 0.083 (0.048) | 0.023 (0.032) |
| Pacific South | 0.009 (0.013) | -0.010 (0.014) | | 0.028 (0.015) | 0.090 (0.046) | -0.015 (0.035) |
| Peninsula | -0.003 (0.013) | -0.008 (0.012) | | -0.030 (0.013)* | -0.074 (0.045) | -0.060 (0.035) |
| **Survey year** |  |  | |  |  |  |
| 2022 | 0.004 (0.009) | -0.013 (0.008) | | 0.009 (0.009) | 0.025 (0.032) | 0.001 (0.024) |
| 2023 | 0.012 (0.009) | 0.002 (0.010) | | 0.016 (0.009) | -0.022 (0.031) | -0.021 (0.020) |
| **Health system supply** |  |  | |  |  |  |
| Public clinics rate | 0.050 (0.033) | 0.021 (0.031) | | 0.041 (0.039) | 0.111 (0.112) | 0.126 (0.069) |
| Private clinics rate | -0.029 (0.074) | -0.137 (0.101) | | 0.031 (0.080) | 0.221 (0.273) | 0.134 (0.209) |
| General practitioners (total) | -0.507 (1.067) | 0.269 (1.547) | | 0.778 (1.233) | -2.985 (3.951) | -3.981 (2.692) |

**Notes.** Reference categories (omitted from the table body) are as follows: **DISLI (deprivation level): Low; Well-being tercile: 1; Marital status: Married; Education: Primary; Smoking status: Never smoked; Region: Pacific North; Survey year: 2021.** Cells report **coefficient (standard error)**, both rounded to **3 decimals**. Rows for **reference categories** are **omitted**; the reference group for each variable is listed in the table footnote. Models are adjusted for the covariates shown. • Scaling: coefficients and standard errors were multiplied by 100 for the following predictors: Public clinics rate; Private clinics rate; General practitioners (total) exceptions (mean glucose models).

**Interpretation.** For **binary outcomes**, coefficients represent **absolute percentage-point differences** in the outcome relative to the reference category (for categorical predictors) or **per 1-unit increase** (for continuous predictors). For **continuous outcomes**, coefficients represent **absolute differences** in the outcome units: **HbA1c** in **percentage points (% )**, **glucose**/**triglycerides**/**total cholesterol** in **mg/dL**, **waist circumference** in **cm**, **BMI** in **kg/m²**, and **eGFR** in **mL/min/1.73 m²**. Rates (e.g., public/private clinics, general practitioners) are interpreted **per 1-unit increase on the reported scale**.

**Abbreviations.** **DISLI** = Density-Independent Social Lag Index; **BMI** = Body mass index; **HbA1c** = Hemoglobin A1c; **eGFR (CKD-EPI)** = estimated glomerular filtration rate (CKD-EPI equation); **BP** = blood pressure; **HDL** = high-density lipoprotein; **SE** = standard error; **CI** = confidence interval. T2D (self-reported) refers to a self-reported physician diagnosis. Prediabetes by HbA1c is defined as 5.7–6.4%. T2D by HbA1c is defined as ≥6.5%. Mean HbA1c (T2D) and Mean glucose value (T2D) denote mean biomarker values among adults with T2D. Controlled T2D (HbA1c, %) is defined as HbA1c <7.0% among adults with T2D. Mean glucose value (uncontrolled T2D) refers to average glucose levels among adults with uncontrolled T2D. Hypertension (self-reported) refers to self-reported physician diagnosis. Controlled hypertension is defined as SBP <130 mmHg and DBP <80 mmHg among adults with hypertension. Overweight + obesity is defined as BMI ≥25.0; Obesity as BMI ≥30.0; and Abdominal obesity as waist ≥90 cm in men or ≥80 cm in women. Metabolic syndrome follows IDF criteria (central obesity plus ≥2 of: triglycerides ≥150 mg/dL or treatment; HDL-C <40 mg/dL in men or <50 mg/dL in women; elevated BP; fasting glucose ≥100 mg/dL). Hypertriglyceridemia is defined as triglycerides ≥150 mg/dL or lipid-lowering therapy. Low HDL-C is defined as <40 mg/dL in men or <50 mg/dL in women. High LDL cholesterol is defined as ≥100 mg/dL. Total cholesterol is defined as ≥200 mg/dL. Impaired renal function is defined as CKD-EPI eGFR <45 mL/min/1.73 m² (stages G3b–G5).

**Statistical significance:** * p < 0.05; ** p < 0.01; *** p < 0.001; no symbol indicates p ≥ 0.05. Asterisks are appended immediately after the closing parenthesis of the standard error.
